# Carbon Surface-Influenced Heterogeneity of Ni and Co Catalytic Sites as a Factor Affecting the Efficiency of Oxygen Reduction Reaction

**DOI:** 10.3390/nano12244432

**Published:** 2022-12-13

**Authors:** Marc Florent, Teresa J. Bandosz

**Affiliations:** Department of Chemistry and Biochemistry, The City College of The City University of New York, 160 Convent Avenue, New York, NY 10031, USA

**Keywords:** porous carbons, Ni- and Co-based catalytic centers, oxygen reduction, surface chemistry, number of electrons transferred

## Abstract

Highly porous carbon black and micro/mesoporous activated carbon were impregnated with cobalt and nickel nitrates, followed by heat treatment at 850 °C in nitrogen. Detailed information about chemistry and porosity was obtained using XPS, XRD, TEM/EDX, and nitrogen adsorption. The samples were used as ORR catalysts. Marked differences in the performance were found depending on the type of carbon. Differences in surface chemistry and porosity affected the chemistry of the deposited metal species that governed the O_2_ reduction efficiency along with other features of the carbon supports, including electrical conductivity and porosity. While dissociating surface acidic groups promoted the high dispersion of small metal species, carbon reactivity with oxygen and acidity limited the formation of the most catalytically active Co_3_O_4_. Formation of Co_3_O_4_ on the highly conductive carbon black resulted in an excellent performance with four electrons transferred and a current density higher than that on Pt/C. When Co_3_O_4_ was not formed in a sufficient quantity, nickel metal nanoparticles promoted ORR on the Ni/Co-containing samples. The activity was also significantly enhanced by small pores that increased the ORR efficiency by strongly adsorbing oxygen, which led to its bond splitting, followed by the acceptance of four electrons.

## 1. Introduction

One of the factors governing the performance of fuel cells is the efficiency of an oxygen reduction reaction, which is determined by the number of electrons transferred as close as possible to four, an onset potential in the most positive range, small Tafel plot slope, and a high current density. In the specific catalytic reactions of oxygen reduction in either acidic or alkane electrolytes H_2_O or OH^-^ are formed, respectively. So far, the most efficient catalysts have been those based on noble metals, and platinum dispersed on various conductive forms of carbons is considered as the benchmark of the catalytic performance [1]. However, high costs of this kind of catalysts directed a search for efficient fuel cell electrocatalysts to transition metals [2,3]_,_ and even to nonmetal catalysts [4,5,6]. While the latter are mainly based on carbon materials modified with various heteroatoms including nitrogen, sulfur phosphorus, and boron, the former approach focuses on the catalytic role of Fe, Ni, Co, or Cu species [2,3]. The emphasis is usually on a catalyst chemical and physical form and on its dispersion on a carbon support. Recently, research efforts have been focused on so-called single atom catalysts [7], SAC, where an atomically dispersed metal is chemically coordinated with nitrogen atoms embedded into the carbon matrix [8]. These materials allow the most efficient utilization of catalytic sites and a provide high stability [9]. For formation of SAC the specificity of a carbon support is a very important feature. Its significance is also demonstrated in a separate line of research dedicated exclusively to carbon catalysts, whose performance has been recently summarized in various reviews [4,5,10,11,12,13,14,15,16]. The advantages of these materials in replacing Pt/C are in a sufficient conductivity and in the relative easiness of the modulation of their electronic structure by an introduction of heteroatoms [17,18,19,20,21,22,23,24,25,26,27,28,29]. A special emphasis has been placed on nitrogen [19,20], either alone or in combination with sulfur [22,23,27], and oxygen [28], which, by providing basic and positively charged sites to the carbon surface, attracts oxygen as a first step of its reduction process. Another important heteroatom for ORR is sulfur [21], which has been found as advancing the reduction process when located in the proximity of nitrogen-containing centers [29].

Another catalytically important feature of the carbon materials is their porosity, which has recently been indicated as advancing the oxygen reduction process [10,30,31,32,33,34,35,36,37,38,39,40,41]. This includes the effect of mesopores as mass transport pores [10] and that of micropores as pseudocatalytic centers contributing to reduction of oxygen through providing a strong adsorption potential [34,35,36,37,38,39,40,41]. Moreover, the higher porosity of carbon is often directly linked to the higher structural defects which were also identified as the catalytic ORR centers [16,42,43].

Carbon blacks are a commodity of a highly conductive nature and they have been commonly used as supports for a high dispersion of noble metal-based catalysts [44,45]. Nevertheless, for this purpose rather nonporous carbon blacks, such as Vulcan, have been explored over their highly porous counterparts [46,47]. However, there are reports on carbon blacks’ direct usage as transition metal catalyst supports [47,48,49,50,51,52] and catalysts themselves [41], especially those modified with nitrogen [53,54]. Even though there are numerous reports on the catalytic effects of transition metals on oxygen reduction on various carbon materials [2,8], carbon blacks deserve special attention not only owing to their high electrical conductivity, but also due to the possibility of utilization of their specific porosity to advance ORR [40,41]. Often that porosity effect might have been overseen when the emphasis of reports was on a direct influence of metal catalysts. Nickel and cobalt are considered as the most suitable catalysts for ORR, and since the works of Bagotzky et al. [55,56], many researchers have reported their positive influence on this reaction; examples are references [48,49,52,55,56,57,58,59,60,61,62,63,64,65,66,67,68,69,70]. It has been proposed that either single metal atoms coordinated with nitrogen embedded to the carbon matrix [57,58,60,61], metal nanoparticles [59], and oxides [64,66], especially Co_3_O_4_ [52,57] and oxyhydroxide [64], can work as the ORR catalysts. On the other hand, there are also reports claiming that these metals work as the ORR catalysts only when coordinated with nitrogen [68].

Recent works on various forms of carbon materials, including carbon blacks, activated carbons, carbon nanotubes, or carbon aerogels modified with cobalt and nickel as ORR catalysts have reported that the number of electrons transferred in alkaline electrolytes can reach four [60]; the onset potential can even be more positive than that on Pt/C [57,58], and the kinetic current as high as 8.1 mA/cm^2^ [60]. When a catalytic action of both metals is considered, cobalt usually outperforms nickel [67] and NiCo alloys, alone or integrated with their oxides, and have shown an exceptional catalytic activity [69,70].

Published reports on Co and/or Ni-containing carbons as the ORR or bifunctional catalysts mainly link the catalytic activity to the content of metal on the surface and its dispersion, presence of nitrogen providing the coordination sites, and form/chemistry of Co and Ni species. Some of them analyze the effects of catalyst precursors [59]. The most common catalyst supports are porous carbons (either activated ones or their synthetic counterparts) and carbon blacks. While the former, doped with N or undoped, provide a developed surface area promoting the high dispersion of catalytic sites, the high conductivity of the latter is considered as an advantage, which also directed these materials to be used as the Pt catalysts supports. What is often not considered as an advantage of carbon blacks is the high surface area of some specific commercial products, and an example is Black Pearl 2000. Even though Black Pearl carbon blacks have been studied as ORR catalysts and supports with some conflicting results [41,44,68,71], and recently the role of oxygen adsorption in pores of the Black Pearl carbon family has been indicated as affecting the efficiency of ORR [40,41], in our opinion, in the results published so far there has not been enough emphasis on researching the role of the carbon black surface in promoting the activity of the Co/Ni catalysts for ORR and in providing synergistic activity. Our inspiration for pursuing this angle of research is in our recent results on the excellent performance of this commodity, either unmodified [41] or modified with nitrogen [72], in the oxygen reduction process. To provide deeper insight into this perspective, we analyzed Co and/or Ni modified Black Pearl 2000 catalysts in parallel with those based on commercial wood-based activated carbon BAX-1500 of a highly developed surface area in a micro/mesoporous structure. The results discussed threw new light not only on the nature of active sites affecting ORR, but also on the role of the carbon support in bringing this kind of complex catalytic activity. Our efforts focus on “old” carbon forms to provide relatively broad conclusions and to emphasize the economic aspects of targeting alternative sources of energy.

## 2. Materials and Methods

### 2.1. Materials

Commercial carbon black from Cabot Corporation (Boston, MA, USA), Black Pearls 2000 (referred to as BP), and wood-based activated carbon from Ingevity (North Charleston, SC, USA), BAX-1500 (referred to as BAX) were used as catalysts supports. While BP was used as-received, BAX pellets were first ball-milled and sieved to a particle size below 212 μm. Cobalt nitrate hexahydrate (Co(NO_3_)_2_∙6H_2_O) and nickel nitrate hexahydrate (Ni(NO_3_)_2_∙6H_2_O) were used separately or combined in a 1 to 1 mass ratio of the salts to introduce metal-base active phase onto the carbon surface. BAX or BP was first impregnated with an aqueous solution of metal salt using a 1:1 mass ratio of carbon to salt (equivalent to 1:0.2 mass ratio of carbon to metal). After drying, the mixtures were heated at 850 °C for 30 min, with a heating rate of 10 °C/min and under a nitrogen atmosphere (100 mL/min). The samples were then washed in a Soxhlet apparatus until constant pH and dried at 120 °C. Two series of three samples, BP-Ni, BP-Co, BP-NiCo and BAX-Ni, BAX-Co, and BAX-NiCo were prepared in this way, where the first term designates the type of carbon (BP or BAX) and the second term the kind of metal incorporated (Ni, Co, or a 1-to-1 mix of Ni and Co).

### 2.2. Methods

#### 2.2.1. Surface Characterization

Nitrogen adsorption isotherms measured on an ASAP 2020 (Micromeritics, Norcross, GA, USA) at −196 °C, after degassing the samples at 120 °C overnight, were used to calculate a surface area, S_BET_ (using the Brunauer–Emmett–Teller theory), and total pore volume V_tot_ (from the total amount of nitrogen adsorbed at relative pressure of 0.98). A volume of micropores, V_mic_, was calculated using 2-NLDFT assuming the pore wall heterogeneity [73]. This method was also used to provide pore size distributions. The volume of mesopores V_mes_ was calculated from the difference between V_tot_ and V_mic_.

Thermogravimetric (TG) and differential TG (DTG) analyses were carried out on an SDT Q600 (TA Instruments, New Castle, DE, USA). The samples were heated up to 1000 °C with a heating rate of 10 °C/min and under a 100 mL/min argon or air flow.

XPS spectra were collected on a Physical Electronics (Chanhassen, MN, USA) PHI 5000 VersaProbe II spectrometer (using an Al Kα X-ray source (50 W, 15 kV, 1486.6 eV) and a take-off angle of 45°). A concentric hemispherical analyzer operated in a constant-pass-energy mode, at 29.35 eV, with a 200 μm diameter analysis area. Multipack software was used to deconvolute the spectra.

Resistivity of the materials was measured using a Keithley 2400 Multimeter (Keithley Instruments, Cleveland, OH, USA) on a 0.5 × 0.45 cm gold interdigitated electrode covered with a thin film of carbon.

TEM images were taken with a JEM2100 electron microscope (JEOL, Tokyo, Japan) operated at a 200kV voltage. It is fitted with an Inca Energy Dispersive X-ray Spectrometer (EDX) system used to obtain the elemental maps.

Oxygen adsorption in solution was measured by recording the concentration of dissolved oxygen in 50 mL water using a Hach (Loveland, CO, USA) IntelliCAL LDO probe upon the addition of 250 mg of the carbon sample.

#### 2.2.2. Electrochemical Measurements

Electrochemical measurements were carried out with a 0.1 M KOH electrolyte (pH = 13). A three-electrode system was controlled with a WaveDriver 40 bipotentiostat (Pine Research Instrumentation, Durham, NC, USA). A rotating ring-disk electrode (RRDE) with a gold ring and a glassy carbon disk (0.1963 cm^2^) coated with a catalyst ink (a thin film on the carbon disk was made of 33.3 μg of a sample and the same amount of Nafion binder) were used as a working electrode, a graphite rod as a counter-electrode, and a silver/silver chloride electrode (filled with 4 M KCl) as a reference electrode. Potentials E_Ag/AgCl_ measured were converted to potentials versus reversible hydrogen electrode, E_RHE_, using the following formula:

(1) $$E_{\mathrm{RHE}}={\;E}_{\mathrm{Ag}/\mathrm{AgCl}}+0.199+0.59\mathrm{pH}$$

Cyclic voltammetry (CV) curves were measured at a scan rate of 5 mV/s after saturation of the electrolyte solutions with either nitrogen or oxygen gas.

Electrochemically active surface area (ECSA) of the samples was estimated from their double layer capacitance (C_DL_), assuming that they have a similar specific capacitance C_S_ equal to that of carbon black, 27.5 µF/cm^2^ [74]:

(2) $$\mathrm{ECSA}=\frac{C_{\mathrm{DL}}}{C_{S}}$$

C_DL_ was calculated from the cathodic (I_c_) and anodic (I_a_) current measured at different scan rate between 5 and 150 mV/s in a non-faradaic region [75]:

(3) $$C_{\mathrm{DL}}=\frac{I_{c}+I_{a}}{2\nu }$$

Linear sweep voltammetry (LSV) curves were recorded at various rotation speeds (ω, between 400 and 2000 rpm) in oxygen-saturated electrolytes while keeping the ring at a constant potential of 1.07 V vs. RHE. They were corrected for any capacitive currents by subtracting the background LSV curves measured in oxygen-free electrolytes.

The kinetic current was calculated from the LSV curves, using the Koutecky–Levich Equation (3) [75]:

(4) $$\frac{1}{I_{D}}=\frac{1}{I_{l}}+\frac{1}{I_{k}}=\frac{1}{{B\omega }^{1/2}}+\frac{1}{I_{k}}$$

where I_D_, I_l_, and I_k_ are the disk, diffusion limited, and kinetic currents, respectively. They were divided by the carbon disk area, 0.1963 cm^2^, to obtain the corresponding current densities J_D_, J_l_, and J_k_.

The onset potential was calculated from the LSV curves measured at 2000 rpm using the unbiased second derivative method [76], and the number of electrons transferred, n, and the percentage of H_2_O_2_ produced from Equations (4) and (5), respectively [75]:

(5) $$n=\frac{4I_{D}}{I_{D}+\frac{I_{R}}{N}}$$

(6) $${\%H}_{2}O_{2}=\frac{200\frac{I_{R}}{N}}{I_{D}+\frac{I_{R}}{N}}$$

where I_D_ and I_R_ are the disk and ring currents, respectively, and N the collection efficiency of the ring-disk electrode is equal to 25.6%.

The Tafel plot of the potential as a function of log(|J_k_|) was built in a region close to the onset potential and the slope was obtained by a linear fit.

The stability of the samples was evaluated by chronoamperometry at the potential of a maximum oxygen reduction current, with a constant O_2_ flow through the electrolyte, for 24 h.

An ethanol crossover effect was tested by measuring the change of a current at the potential giving a maximum oxygen reduction current, upon addition of 2.5 mL of methanol into 60 mL of the electrolyte solution.

## 3. Results and Discussion

**Figure 1:**
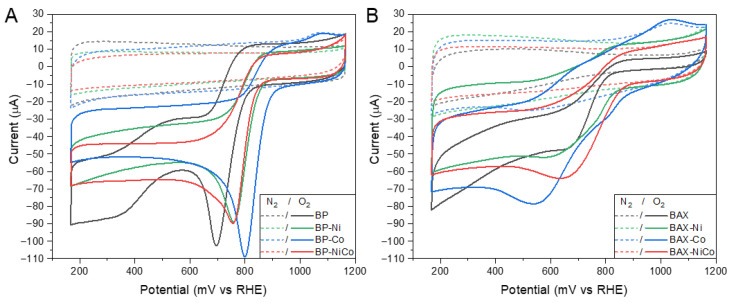
CV curves in oxygen and nitrogen saturated electrolytes for the BP (**A**) and BAX (**B**) series.

Figure 1 presents the comparison of the CV curves collected in the oxygen and nitrogen saturated electrolytes, where oxygen reduction humps are clearly seen with differences in their intensity and the positions of hump minimum/maximum currents. While the oxygen reduction process is better defined for the BP series, a capacitive behavior is more pronounced for the BAX series.

**Figure 2:**
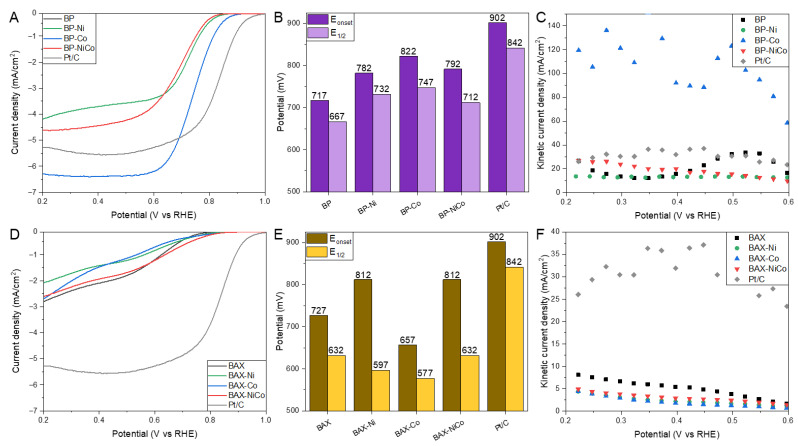
Current density for the BP series samples (**A**) and the BAX ones (**D**); onset potentials for the BP series samples (**B**) and the BAX ones (**E**); the kinetic current densities calculated using Koutecky–Levich Equation for the BP series samples (**C**) and the BAX one (**F**).

As expected, LSV curves (Figure S1 of the Supplementary Information) show that the current depends on a rotation rate, especially for the BP series, which is typical for a mixed kinetic-diffusion control process. Current densities at 2000 rpm are compared in Figure 2. The modification of BP with metals significantly improved the performance (Figure 2A). While the LSV curve of the initial BP sample exhibits typical two waves characteristic for porous carbons [32], the modification with metals markedly changed the shapes of the LSV curves, making them similar to that measured on Pt/C. Interestingly, the current density measured on BP-Co exceeds that of the platinum-based catalyst of about 1 mA/cm^2^ (6.3 vs. 5.3 mA/cm^2^). That current density is higher than those measured on carbon blacks modified with cobalt reported in the literature [52,71]. For example, on Black Pearls 2000 with deposited Co_3_O_4_ the current density reached 5.6 mA/cm^2^ [52], and on the same carbon black modified with cobalt acetate-4.5 mA/cm^2^ [71]. A similar value of the current density (6.18 mA/cm^2^) was reported on carbon black with deposited carbonized Co^2+^, Zn^2+^, and 2-methylimidazole [57]. The current density of BP-NiCo is between those for the Co- and Ni-modified samples, which might suggest the predominant effect of the cobalt species on the ORR activity on these samples. The onset potential is reported as that calculated from the unbiased second order discrete differentiation method (SODDM) [75]. For the sake of discussion, we also reported a half wave potential (Figure 2B). For BP-Co, which is the best-performing sample, it is 80 mV less positive than that on Pt/C, and the other two samples show values lower of 115 mV when compared to Pt/C, with BP-Ni as the worst-performing catalyst. Even though, due to various approaches used in the literature to deliver onset potential values discussed in detail in Ref. [76], the direct comparison of this quantity reported might be biased, it is important to mention that the introduction of Co species led to the onset potential of ORR up to 85 mV more positive than that on the unmodified sample. Liu et al. reported the onset potential of 860 mV on amide grafted and modified with cobalt Vulcan XC-72, while on Black Pearls 2000 with CoO_x_ only 750 mV was measured [71]. Among other results reported in the literature, on nickel porphyrin-modified carbon fiber the onset potential was 820 mV [77], on tetraphenolphthalein cobalt (II) phthalocyanine polymer modified multiwalled carbon nanotubes it was 803 mV [78], and on atomically dispersed cobalt on N-doped carbon frameworks - 870 mV [60]. In terms of the kinetic current density calculated using the Koutecky–Levich Equation (Figure 2C), BP-Co shows an exceptional performance, reaching ~130 mA/cm^2^, which is much higher than the kinetic current density measured on Pt/C (~35 mA/cm^2^). The results obtained on BP-Co are better than those reported in the literature on Co-modified carbon, [52,57,71,78] and even on those where SAC were directly targeted [15,59,79,80].

The LSV curves for the BAX series show marked inferiority in the current density compared to that measured on the BP series (Figure 2D). It is interesting that initially BAX shows the highest current density and highest kinetic current density (Figure 2F), and the modifications with metal have detrimental effects on these quantities. Among the modified samples, Co shows the highest current density; however, it reaches only 2.6 mA/cm^2^. For this series, the comparison of the onset potential values points to the modification with Ni as the most efficient one and the onset potential on these samples being 90 mV less positive than that on Pt/C (Figure 2E). Nevertheless, the two-wave shape of the LSV curve is still preserved and the current density is low, indicating that even though the process can start at the relatively positive potential value, the ORR process encounters serious limitations on these materials. This low current density led to marked differences between the onset potential calculated using the unbiased SODDM method [76], and the potential at a half wave. Interestingly, the onset potentials of both initial carbons are quite similar to each other.

**Figure 3:**
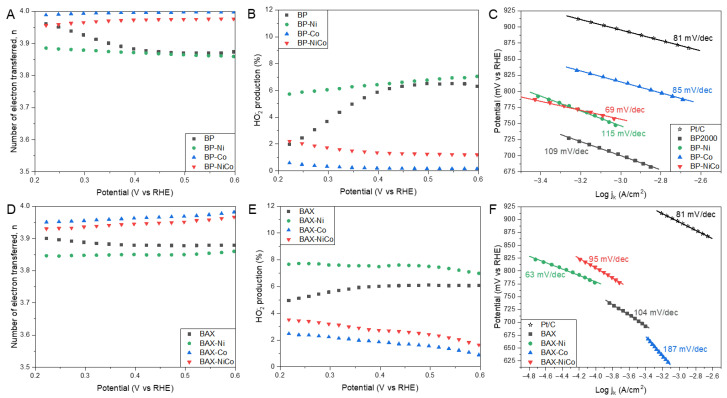
The number of electrons transferred on the BP series samples (**A**) and the BAX ones (**D**); percent of HO_2_^−^ formed in the BP series samples (**B**) and the BAX ones (**E**); Tafel plot for the BP series samples (**C**) and the BAX ones (**F**).

The comparison of the number of electrons transferred is presented in Figure 3A,D. Even though for all samples n values can be considered as reaching 4 (between 3.85 and 4), indicating the high efficiency of the reduction process, in this case the BP series also outperforms the BAX one. Nevertheless, for both series, the best results are obtained on the cobalt-containing samples, and mixing nickel and cobalt (= less cobalt) worsened the performance even when compared to that of the initial samples. Values of n close to four were reported on many carbon-based samples containing cobalt and modified by various approaches [15,52,81], but it is important to mention that the performance of our modified BP samples is better than that reported by Liu et al. [71] and by Goubert-Renaudin and Wieckowski [68] on the same catalyst support. It is also important to bring up that the amount of peroxide formed on the cobalt-containing samples (Figure 3B,E) is even smaller than that on Pt/C and the highest amount formed on BAX-Ni reaches only 8 %, which is significantly lower than on other carbon samples reported with similar number of electrons transferred [81].

The Tafel plot slopes’ values are linked to the kinetics of an electron transfer [24]. Figure 3C,E,F show the Tafel plots with their calculated slopes. For Pt/C the slope of 0.81 mV/dec was found and BP-Co exhibits a very similar behavior. The modification improves the kinetics of the ORR process on both series of carbons. The worst kinetics are on the BAX series and especially on BAX-Co, and the best, even better than that on Pt/C, are on BAX-Ni. In terms of the kinetics of the electron transfer, modified carbon black tested by Cheng et al. [52] showed better results, which might be attributed to the additional modification of their catalyst support by nitrogen.

**Figure 4:**
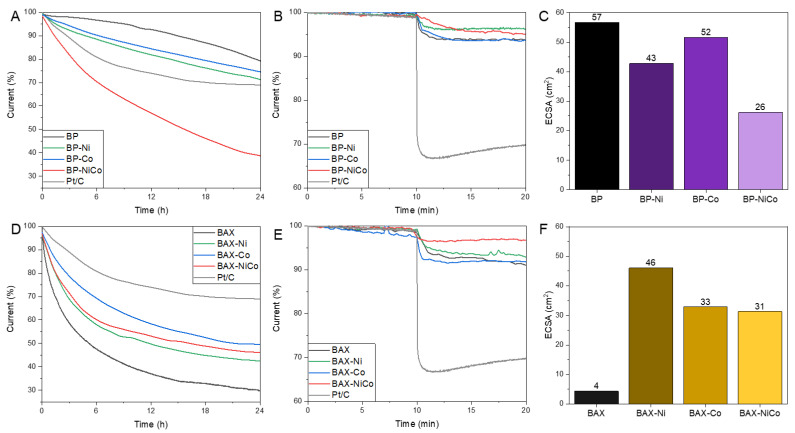
Stability of the BP series (**A**) and of the BAX ones (**D**); stability to methanol cross-over to the BP series (**B**) and BAX ones (**E**). ECSA of the BP (**C**) and BAX series (**F**).

The stability of the catalysts is compared in Figure 4A,D. While all BAX samples are less stable than is Pt/C, in the case of the BP series, better performance than that of Pt/C was found for the single-metal-modified samples, but their stability is worse than that of the initial BP. Interestingly, in the case of the BAX series, the modification increased the stability compared to that of the initial sample, which might be linked to exposing carbon to a higher temperature than that used in the synthesis of original BAX (600 °C). The difference between the BP and BAX series are likely caused by the differences in susceptibility of their carbon matrices to oxidation by formed H_2_O_2_. Testing of the effect of the methanol cross-over shows the significantly higher stability of all samples tested than that of Pt/C (Figure 4B,E).

The measured ECSA are collected in Figure 4C,F. This parameter is considered as reflecting the specific activity of a catalyst, and the highest values measured for the BP and BAX series are those on BP-Co and BAX-Ni, respectively, which reflects the trend found in the onset potential values. Mixing Ni and Co decreases ECSA, which is also the observed effect on other ORR process descriptors. Interestingly, initial BP has higher ECSA than the modified samples while a totally opposite trend was found for the BAX series. This might indicate that for the initial BP sample, the high electrochemical activity should be linked to totally different factors than those governing the modified BP samples, and those factors were discussed elsewhere [27,30,31,32,33,34,35,36,37,38,39,40,41]. In the case of the BAX series, the modification with metals introduced highly active in ORR surface sites.

The presented above results of the electrochemical tests clearly show the effects of a carbon support on the performance of the catalysts. This performance is expected to be markedly influenced by resulting chemistry and the porosity of the catalysts influenced by the support. An important factor in our consideration is that the amounts of Ni and Co salts originally used for the modifications and the conditions of the treatments were the same for both series of samples. Therefore, any differences/trends in the surface properties should be linked to the effects of the carbon support and the interactions of their surfaces with deposited Co or/and Ni nitrate salts, and to the complex effects of the applied heat treatment on the resulting surface features. Following the general consensus, a surface rich in carboxylic groups is expected to increase the dispersion of metals, and the thermal decomposition of nitrates into NO_x_ might oxidize/activate the surface and cause even some degree of mass loss/volatilization (as CO/CO_2_) in the case of supports with a low level of carbon ring condensation. It might also result in the development of an additional porosity. The deposition of salts, especially in large agglomerates, might also block some pores and decrease a pore volume and surface area determined by the N_2_ molecule.

To understand the direction of surface chemistry and porosity changes that affect the final catalytic performance, we will first analyze the differences in the nature of the metal species deposited on the carbon surfaces. Even though these carbons were arbitrarily chosen owing to the specific micro/mesoporosity resulting in the high BET surface areas, they still differ significantly in other surface features. Burning carbon samples in air provides the information on the content of an inorganic matter, which in our case is considered to contain in majority nickel and/or cobalt oxides, remaining as a residue at 1000 °C. The comparison of TG and DTG curves measured in air is presented in Figure S2. Based on the preparation procedure, the samples should contain ~17% of metals assuming that released nitrogen oxides did not cause an extensive burn-off of the carbon matrix. This seems to be the case for the BP series where ~20% of metal oxides are detected for all samples. The situation is different for the BAX series, where the content of metal oxide exceeds the theoretical amount and is about 5% higher than in the BP series. A plausible explanation is more extensive burn-off of the BAX support resulting in the removal of carbon as CO_2_/CO during the thermal treatment in nitrogen. Obviously, more metal species on the surface of the BAX series did not lead to the higher catalytic activity.

**Figure 5:**
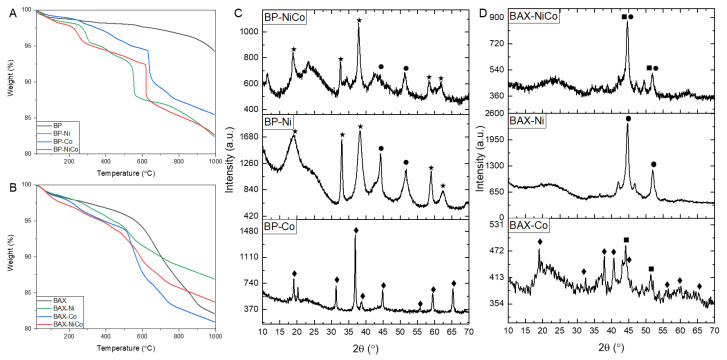
TG curves in argon for the BP series (**A**) and the BAX ones (**B**); XRD of the BP series (**C**) and the BAX ones (**D**); ■, ● Co and Ni cubic Fm-3 m, respectively, ★ Ni(OH)_2_ hexagonal P-3m1, ◆ Co_3_O_4_ cubic Fd-3 m.

Some information about the differences in chemistry of the metal species can be provided by the thermal analysis conducted in argon, and the results, as TG and DTG curves, are presented in Figure 5A,B and Figure S3, respectively. Thermal events related to the decomposition and reduction of surface species by carbon are seen as weight losses on the TG curves or peaks on the DTG curves. Compared to the initial BP sample, the modified samples of this series exhibit a very chemically heterogeneous surface (Figure 5A). The first weight loss between 200 and 300 °C is linked to the dehydroxylation of either Co(OH)_2_ or Ni(OH)_2_, which leads to the formation of corresponding oxides that are reduced to metal between 500 and 700 °C. This process is reflected by sharp decreases in the weight. In the case of BP-Co, two steps are visible in this temperature range, suggesting the presence of cobalt in a higher oxidation state than +2, which was first reduced to CoO and then the reduction of the latter took place. The weight loss pattern for BP-Co suggests that hydroxide is present in a very small amount, or the particles of cobalt oxides are rather small and their reduction takes place gradually between 300 and 600 °C, with larger particles reduced at higher temperatures. The shape of the TG curve for BP-Ni rather suggests the less heterogeneous distribution of sizes than that for BP-Co, since a well-defined reduction event is detected at ~400 °C. The weight loss of BP-NiCo is apparently influenced by some mixed Co/Ni forms. For the BAX series, the comparison of the weight loss pattern of the modified samples with that of the initial sample should be performed only up to 600 °C, since it is the highest temperature that carbon was exposed to during its production. Gradual weight losses for BAX-Ni and BAX-NiCo suggest the presence of oxygen-containing particles of various sizes. The BAX carbon matrix reduces the majority of oxides between 500 and 800 °C and a two-step weight loss in this temperature range implies that some Co might be in a +3 oxidation state. In fact, the weight loss for BAX-Ni, and especially that for BAX-Co, resemble that for BP-Co between 600 and 800 °C; however, the reduction of the former sample starts to occur at lower temperatures, ~500 °C. The results of the thermal analysis in argon suggest that the particles of metal species/oxides on BP are larger than those on BAX, especially in the case of cobalt, and the particle sizes in the case of BAX are more heterogeneous in their sizes’ range.

X-ray diffraction experiments bring information about the speciation of the crystallographic phases and on the sizes of the crystals on the surface of the carbon supports (Figure 5C,D). For the BP series, the highest crystallinity level of the deposited species is seen for BP-Co, whose diffractogram shows show many sharp peaks representing Co_3_O_4_ with large crystals sizes of 27.5 nm, as calculated from the Scherrer Equation. The BP-Ni crystallographic phases are more chemically heterogeneous and broader than in the case of BP-Co diffraction peaks, and are assigned to Ni(OH)_2_ and metallic nickel crystals with sizes of about 6.2 and 6.9 nm, respectively. The presence of hydroxide in the case of BP-Ni is consistent with the sharp weight loss seen at ~300 °C on the TG curve of this sample measured in argon (Figure 5A). When Ni and Co are deposited simultaneously on the surface of the BP sample, the resulting crystallographic phases are the same as in the case of BP-Ni but the sizes of crystals are slightly larger, where Ni(OH)_2_ size is ~12.2 and that of Ni metal particles of 10.4 nm.

The diffraction patterns of the BAX series totally differ from those of the BP samples. Even though some crystals are present on the surfaces, the results suggest that the inorganic species are more dispersed than are in the latter series, and various phases of hydroxides and oxyhydroxides were identified [82], especially on BAX-Co and BAX-NiCo. Their presence might explain a gradual reduction of the surface seen on the TG curves measured in argon (Figure 5B). On both BAX-Co and BAX-Ni, corresponding metal particles are detected with sizes of 5.2 nm and 11.5 nm, respectively. These crystals are also present in BAX-NiCo and their sizes are about 14.6 nm. On BP-Co small crystals of Co_3_O_4_ exist. The most dispersed/amorphous inorganic species are on BAX-Ni.

**Figure 6:**
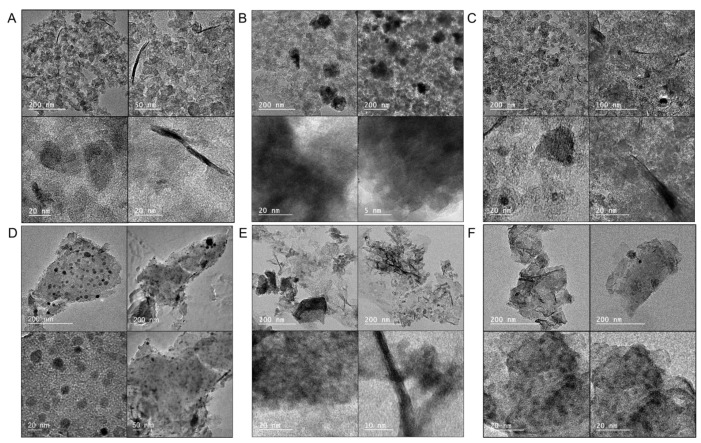
TEM images of BP-Ni (**A**); BP-Co (**B**); BP-NiCo (**C**); BAX-Ni (**D**); BAX-Co (**E**); and BAX-NiCo (**F**).

**Figure 7:**
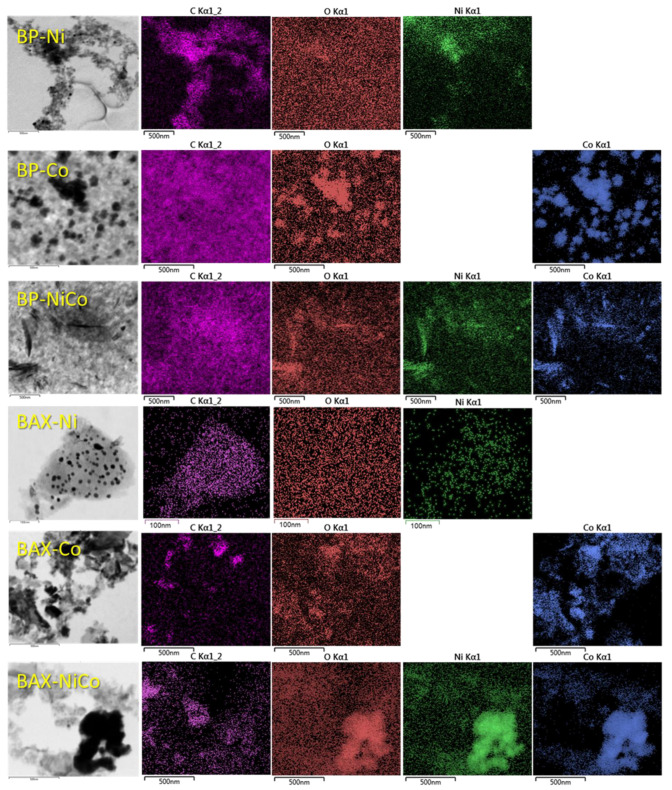
EDX maps of the BP and BAX series of samples.

The trends obtained from the TA and X-ray analyses are confirmed in the TEM images of the samples presented in Figure 6, where the metal-containing particles on the surface of the BAX series are much smaller than those on BP, and their dispersion is much higher on the former samples. In the case of the BP series, the highest dispersion of the metal-containing species was found on BP-Ni, and this is consistent with the differences in the diffraction patterns (Figure 5D). These results are confirmed by the EDX maps (Figure 7). The comparison of cobalt and oxygen signals supports the existence of the metal nanoparticles on both BP-Co and BAX-Co. In the case of BP-NiCo, even though there is a strong indication of the coexistence of both metals in Co+ Ni +O configurations, the analysis of the maps still suggests that the particles of both metals are deposited on the carbon surface.

**Figure 8:**
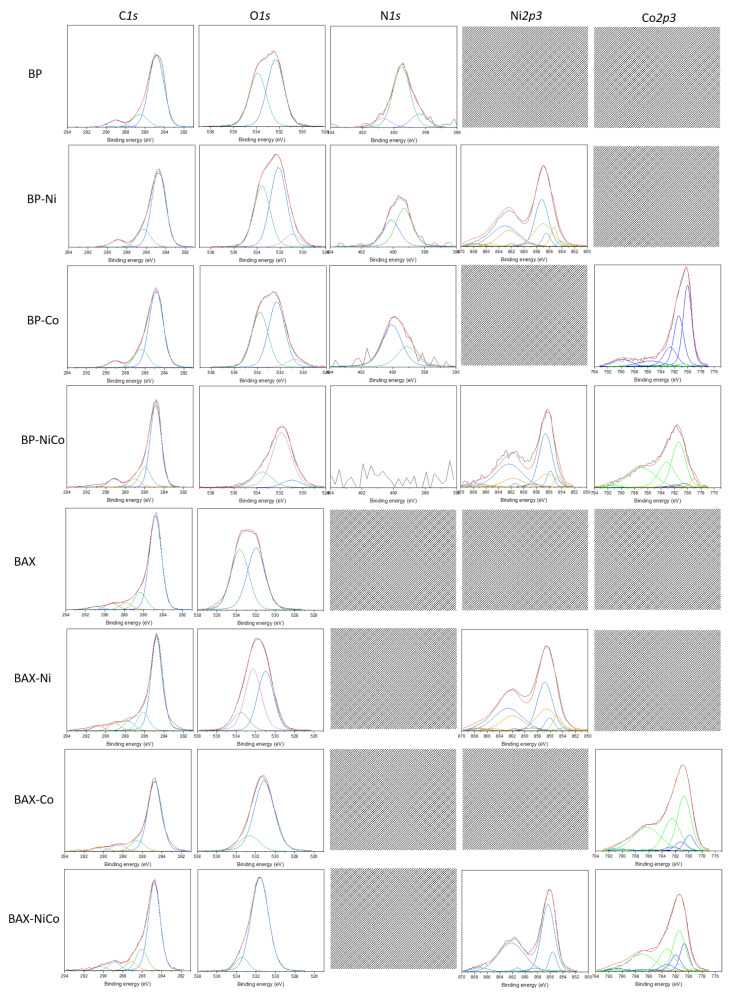
Deconvoluted C 1s, O 1s, N 1s, Co 2p and Ni 2p core energy level spectra (the lines of different colors are used for better visibility of the spectra components).

**Figure 9:**
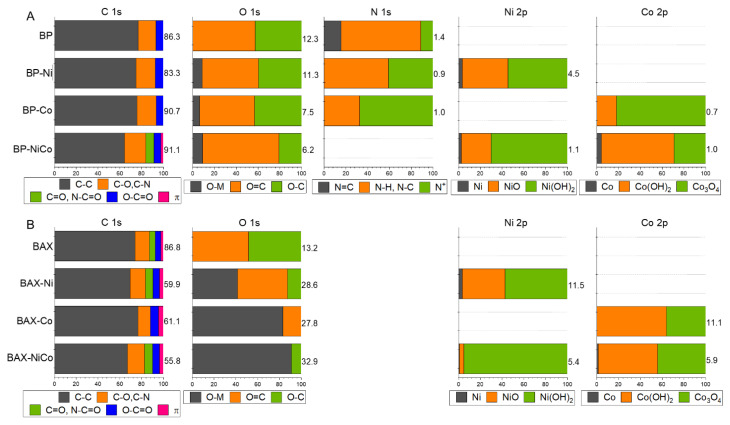
Atomic % of the elements on the surface (as numbers on the right of each segment)) and the contributions of various bonds from the deconvolution of C 1s, O 1s, N 1s and S 2p core energy level spectra (in % contribution on x axes (from 1–100 %) for the BP series (**A**) and BAX series (**B**)).

To assess the surface chemical environment in more detail, an XPS analysis was performed and the results are collected in Figure 8 and Figure 9, and in Table S1 of the Supplementary Information. Even though both initial samples have similar contents of oxygen, the thermal treatment with nitrates led to a marked increase in the surface oxygen for the BAX series and to a decrease for the BP series, which might be associated with differences in the oxidation states of both metals, those of the carbon matrices, and the sizes of the oxygen-containing particles. The deconvolution of O 1s core energy level spectra indicates that the vast majority of oxygen in BAX is in oxides with some contributions from hydroxides and carbon–oxygen bonds, while for the BP series oxygen is mainly associated with the metal phase and in the form of hydroxides. The small detected amount of oxygen in the BP series might be linked to the presence of the metal particles or to the existence of the large particles of the oxygen-containing inorganic phase.

As for oxygen, the BAX series also exhibits much more Ni and Co on the surface than does the BP series. As mentioned above, this is likely related to the larger particles of the metal species in the case of BP series. A 7 nm limit of the XPS surface analysis is not able to account for all metals and oxygen present in these particles, leading to their underestimated amounts. Ni 2p3/2 and Co 2p3/2 core energy levels were deconvoluted using multiplets based on the deconvolution approach of Biesinger et al. [83]. A combination of Ni, Ni(OH)_2_ and NiO was used to deconvolute Ni 2p3/2, and a combination of Co, Co(OH)_2_, and Co_3_O_4_ was used to deconvolute Co 2p3/2, keeping their relative positions, FWHM, and area ratios (as given in Ref. [83]) fixed. Consistently with the X-ray analysis, both Ni and Co metallic particles are clearly detected on the surfaces of BP-Ni and BP-NiCo. While on the BAX carbon sample modified with either Ni or Co and on corresponding BP the metallic particles are present, on BP-Co mainly Co_3_O_4_ is detected by XPS, in agreement with the X-ray results. Some small contribution of Co(OH)_2_ is also detected and it explains the lack of well-defined weight loss on the BP-Co TG curve associated with dehydroxylation and two well-defined steps associated with the reduction of oxides. The chemical distributions of Ni on the BP and BAX samples are similar, but those of cobalt markedly differ between the series and BAX-Co, BAX-NiCo, and BP-NiCo have more Co(OH)_2_ than Co_3_O_4_, making BP-Co an outlier in terms of the Co_3_O_4_ content. On both BP-NiCo and BAX-NiCo hydroxides are in the predominant amounts. It is important to mention that on the BP samples nitrogen is detected in about 1 at.% and upon the treatment, its distribution changes and more nitrogen becomes incorporated to the aromatic rings, especially in the case of BP-Co. Regarding the carbon content on the surface, an increase in the detected relative content of carbon on BP can be explained by the presence of the discussed-above large particles of the metal species. Nevertheless, an increase in the contribution of carbon–oxygen bonds in the case of BAX supports mentioned-above oxidation and burn-off of the BAX carbon matrix by released NO_x_.

Owing to the similarity in preparation, the discussed-above differences in the chemistry of the deposited metal species and in their distribution on the surfaces are expected to be mainly governed by the chemical features of the carbon matrices, which were discussed in detail elsewhere [84]. Here, for the sake of comparison, we reintroduce important information on carbon surface pH, the nature of oxygen groups, and density of those group on the surface of mesopores, where dissociating groups are expected to exist. These features are anticipated to affect chemistry most. Even though both carbons are micro/mesoporous (Table 1), the surface in mesopores composes 38% of the total surface area for BAX and 26 % for BP. Taking into account that on the surface of BAX and BP 1.42 and 0.41 mmol/g of dissociating groups are present (0.71 and 0.19 mmol/g of carboxylic groups), respectively, there are much more groups per unit surface area in the case of BAX than BP (an order of magnitude difference in an average density). Thus, these groups increase the dispersion of the metal cations via cation exchange process and polar interactions. With the same amounts of nitrates needed to be distributed through the surfaces, or even more in the case of the modified BAX samples, this difference leads to the higher density of the metal species and their smaller sizes on the surface of BAX than on BP. This is in agreement with the results of the thermal analysis, XRD, and XPS discussed above. The small sizes of the deposited nitrate particles are readily reduced by the carbon surface during the preparation heat treatment up to 850 °C, forming the metal nanoparticles on the BAX sample. They might even coalesce due to the surface oxidation/decomposition of oxygen groups, forming the well-defined crystals. The relatively large particles of Co_3_O_4_ visible on the surface of BP-Co are formed by oxidation of CoO in large “islands”, existing due to the scarcity of oxygen-containing dissociating group, by NO_x_ from the decomposition of nitrates. The metal contents in the BP series suggest that burn-off/activation of the surface during the preparation heat treatment occurred to a rather small extent. Highly dispersed Co(OH)_2_ formed on the functional groups remains on the surface, as a result of the contact of CoO with ambient air/moisture. In the case of BP-Ni, NiO from the decomposition of nitrates cannot undergo oxidation and remain on the surface in its original form or as Ni(OH)_2_. Here, the highly dispersed small particles of the nickel species are reduced to nickel metal. This is because NO_x_ is able to oxidize only carbon, and formed carbon monoxide is a reducing agent to NiO. In the case of BAX, generally much smaller particles of nitrates were deposited on the surface and therefore the crystallographic phases are not well-defined. Here, Co_3_O_4_ is formed in a much smaller extent not only due the acidic nature of that carbon (pH of BAX is 4.73 vs. 7.98 of BP [84]) but also due to the preference of NO_x_ to oxidize the carbon matrix, as indicated by the TA analysis in air. Interestingly, the surfaces of BP and BAX led to the same extent of the contributions of nickel species, although the Ni(OH)_2_ and NiO particles are much smaller on the surface of BAX and the metal particles are smaller on BP. In the case of mixed metal samples, nickel is present mainly as hydroxides, as also detected by X-ray diffraction. Cobalt is also in majority as Co(OH)_2_; however, it exists in a highly dispersed form. The small particles of Co_3_O_4_ are also present and their contribution is slightly higher on BAX-NiCo than on BP-NiCo.

**Figure 10:**
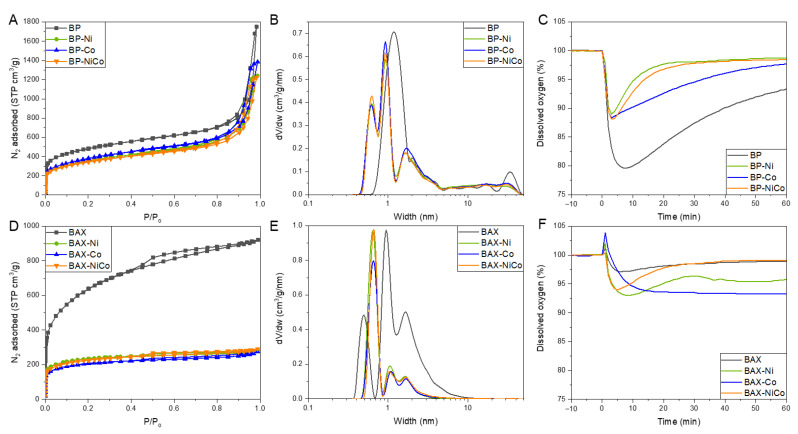
N_2_ adsorption isotherms for the BP series (**A**) and the BAX ones (**C**); pore size distributions for the BP series (**B**) and the BAX ones (**D**); adsorption of oxygen from solution on the BP series (**E**) and the BAX ones (**F**).

The analysis of porosity of the materials tested might bring information not only on the dispersion of the metal phase and on the accessibility of the pores for the electrolyte with dissolved oxygen, but also on the activation/burn off processes taking place during the sample preparation, as speculated above. The measured nitrogen adsorption isotherms and calculated from them pore size distributions and presented in Figure 10A,D. The calculated parameters of the pore structure are collected in Table 1. While the amounts of N_2_ adsorbed on the modified BP samples decreased only slightly compared to that on the initial sample, for the BAX samples a significant decrease in the nitrogen uptake was found. As seen in Table 1, the surface area of modified BAX decreased almost three-fold compared to that of the initial sample. A marked increase in the volume of ultramicropores (V_<0.7_) supports surface activation by NO_x_. On the other hand, over a 50% decrease in the volume of micropores indicates that the deposited species blocks an access of the probe nitrogen molecule to the small pores. The biggest decrease was found in the volume of mesopores and it might be caused by the deposition of the metal species on their surface functional groups, decreasing mesopore sizes markedly. The increase in the volume of ultramicropores is greater for BAX-Ni than that for BAX-Co, which supports the strong oxidation effects of NO_x_ on the carbon matrix of BAX-Ni, as compared to that on the latter sample. In the case of BP samples the porosity of the modified samples is similar, although the nickel-containing sample still exhibits a slightly greater decrease in the surface area compared to that of BP-Co. In these samples some ultramicropores were formed, which could be due to the deposit of relatively large inorganic particles in supermicropores and in mesopores, causing a decrease in their sizes. Moreover, some slight effects of activation by NO_x_ cannot be crossed out.

The pore size distributions presented in Figure 10B,E show the narrowing of the small pores in the BP samples upon the modifications, with a bimodal distribution in the range of micropores. Large mesopores of the BP sample with sizes of about 30 nm significantly decreased in their volume. BAX carbons, although micro/mesopores, have sizes of mesopores reaching only 10 nm and this spatial constraint can be responsible for a marked decrease in their sizes due to the deposition of the inorganic species. For this carbon the ultramicropores increased in size with a marked increase in their volume. Overall, the resulting sizes of ultramicropores are much smaller in the case of the BP samples, and they might favor oxygen adsorption. A large volume of mesopores with the inorganic particles of polar nature promote the transport of the electrolyte with dissolved oxygen to the catalytic centers located on the surface/in small pores.

Another factor to consider as affecting the efficiency of the ORR process is the conductivity of the samples which significantly differs between the two categories of carbons tested. The BP series is much more conductive than the BAX series (measured resistivity for BP, BP-Ni, BP-Co, and BP-NiCo is 11 Ω, 12 Ω, 10 Ω, and 10 Ω, respectively, while that for BAX, BAX-Ni, BAX-Co, and BAX-NiCo is 1.4 MΩ, 0.2 kΩ, 0.3 kΩ, and 9 kΩ, respectively) and this certainly is related to the level of carbon aromatization/graphitization and the nature/amount of oxygen groups. The increased graphitization level of the carbon matrix by the heat treatment at 850 °C combined with the deposition of the metallic particles might explain an increase in the conductivity of BAX-Ni and BAX-Co, which positively affects the performance of these catalysts.

The trend in oxygen adsorption from its saturated solution upon the addition of the catalyst is presented in Figure 10D,F. As expected, based on the distribution of the pore sizes and their volume, there are not marked differences in the maximum of the amount of oxygen adsorbed within series. The BAX samples adsorb less oxygen than do the BP ones, which might contribute to their overall lower catalytic activity [41], and which is in agreement with the differences in the volume and sizes of ultramicropores. Interestingly, the differences are seen in the kinetics of the process where BP-Co and BAX-Co show prolonged adsorption of O_2_ upon its time-dependent dissolution in water. This might be related to the small sizes of cobalt Co^+2^ particles (other than Co_3_O_4_) on these samples, which might be undergoing oxidation to Co_3_O_4_, and those in situ formed catalytic centers (also during the ORR process) might be responsible for the higher stability of these samples compared to their Ni-containing and BP-based counterparts.

The analysis of the differences in the surface features of our samples that are indicated in the literature as important to ORR, and discussed above, allow us to provide a plausible explanation of not only the good catalytic activity of the BP series and especially that of BP-Co, but also to suggest the surface features of the BAX series which result in its inferior behavior. The conductivity of the BP series is certainly their important asset, elevating the current compared to that on the BAX series. Another important feature of the BP series is the presence of nitrogen on the surface. Even in small amounts, that nitrogen, when incorporated to the carbon rings was indicated as enhancing ORR [12,17,19,20,28,34,35,38,42,52,72]. The XPS results showed that the thermal treatment in the presence of metallic species increased the contribution of this specific nitrogen, and thus a nitrogen coordination to metals and the creation of single metal catalytic centers cannot be crossed out [77,78,81]. The BP series have also much smaller ultramicropores than those in the BAX series and this factor is expected to contribute to ORR via strong adsorption of oxygen, followed by its bond splitting [27,34,35,36,37,38,39,40,41]. Additionally, this factor might also contribute to the better performance of the initial BAX carbon than some of its modified counterparts, owing to its much higher porosity. A support for the role of micropores is also the larger number of electrons transferred for all BP samples than those for BAX.

Nevertheless, among the BP samples marked differences in the performance still exist, and BP-Co has been found to be the best catalyst, with the current density higher than that on Pt/C. Since the porosities and conductivities of the BP series are similar to each other, that effect can only be linked to the chemistry of the metal species deposited on the surfaces, in addition to the presence of those hypothesized above catalytic centers based on metal–nitrogen bonds. Since the analyses indicated that Co_3_O_4_ is the predominant species on the surface of BP-Co, we link the excellent performance of this sample to these species, which were also reported in the literature as catalytically active [52,57]. Support for the hypothesized effect of single metal atoms in coordination with nitrogen is the second-best performance of BP-Ni, and these N-M coordinated metals are also expected in the case of nickel modification. The highly dispersed metal particles might also bring the catalytic activity to the Ni-containing samples [85].

As also observed for the BP series, the BAX samples do not differ markedly in the porosity, and the difference in the performance between the samples in this series is expected to be predominantly governed by their surface chemistry. Here, the performance of both nickel-containing samples, BAX-Ni and BAX-NiCo, in terms of the onset potential is similar to that of the BP series, and taking into account all other features of these samples bringing catalytic disadvantages, this suggests that for this quantity the chemistry of the catalytic species is a predominant factor. In addition, the results imply here that the predominant feature governing the onset potential could be highly dispersed nickel and cobalt metal nanoparticles [59,83]. Moreover, the overall performance of BAX-NiCo in terms of the onset potential is better than that of BP-NiCo owing to its higher content of the Co_3_O_4_ catalyst; however, on this carbon the catalytic action of these species is inhibited owing to overpowering effects of other factors contributing negatively to the efficiency of these series of carbons. The catalytic activity of Co_3_O_4_ has been previously linked to the coupling between the carbon surface and this species [86]. Moreover, the specific facets of the crystals might also play a role, as indicated by Liu et al. [87] based on the DFT calculations of the Co_3_O_4_ activity in an acidic electrolyte.

## 4. Conclusions

The results collected show the significant effect of carbon matrix properties on the surface features of the catalysts, and thus on the catalytic performance of Ni- and/or Co-modified carbons influenced by them. The exact same modification procedure resulted in differences in the chemistries of the deposited species, in the sizes of the particles, and in the porosity of the materials. A more oxidized carbon surface, owing to the high density of oxygen groups attracting metal salts during impregnation, led to the high dispersion of metal species of small particle sizes, and thus promoted the formation of metal nanoparticles thorough reduction process, especially in the case of the modification with nickel, where the metal particles were found as promoting ORR. When cobalt was used as a modifier, Co_3_O_4_ was formed and it provided excellent catalytic activity, especially for the BP carbon sample whose surface was poor in dissociating groups. In that case an oxidizing action of NO_x_ was directed mainly to oxidize cobalt and to form spinel Co_3_O_4_. The ORR catalytic efficiency of highly porous carbon black modified with cobalt was only inferior to Pt/C in the slightly lower onset potential, while the current density was much higher than that on Pt/C and the number of electrons transferred reached four with a high catalytic stability. The excellent performance of that carbon black was also linked to its high electric conductivity and porosity. The latter, by the high volume of ultramicropores, enhanced oxygen adsorption and thus provided a pseudocatalytic action complementing the overall influence of catalytic metal-based sites, and especially that of the Co_3_O_4_ particles. These results show promise in the application of commodity carbon blacks as ORR catalysts.

## Figures and Tables

**Table 1:** The parameters of pore structure.

Samples	S_BET_ m^2^/g	V_tot_ cm^3^/g	V_<0.7_ cm^3^/g	V_mic_ cm^3^/g	V_mes_ cm^3^/g	V_mes_/V_tot_ %
BP	1609	2.705	0.001	0.532	2.173	80
BP-Ni	1201	1.919	0.063	0.361	1.558	81
BP-Co	1295	2.141	0.066	0.388	1.753	82
BP-NiCo	1161	1.889	0.067	0.356	1.533	81
BAX	2158	1.424	0.079	0.725	0.699	49
BAX-Ni	741	0.436	0.141	0.321	0.115	26
BAX-Co	658	0.425	0.113	0.272	0.153	36
BAX-NiCo	724	0.451	0.119	0.294	0.157	35

S_BET_—surface area, V_tot_—total pore volume, V_<0.7_—volume of ultramicropores, V_mic_—volume of micropores, V_mes_—volume of mesopores and V_mes_/V_tot_—mesoporosity degree.

## Data Availability

Raw data available upon request.

## Supplementary Materials

The following supporting information can be downloaded at: https://www.mdpi.com/article/10.3390/nano12244432/s1, Figure S1: Ring and disk currents at different rotation (0–2000rpm) of the different samples: BP (**A**), BP-Ni (**B**), BP-Co (**C**), BP-NiCo (**D**), BAX (**E**), BAX-Ni (**F**), BAX-Co (**G**) and BAX-NiCo (**H**); Figure S2: TG (**A**,**C**) and DTG (**B**,**D**) curves measured in air of the BP series (**A**,**B**) and of the BAX series (**C**,**D**); Figure S3: DTG curves measured in argon of the BP series (**A**) and of the BAX series (**B**); Table S1: the atomic content of elements on the surface (in bold) and the deconvolution results of collected core energy level spectra.
