# Supplementary material for: Carbon Surface-Influenced Heterogeneity of Ni and Co Catalytic Sites as a Factor Affecting the Efficiency of Oxygen Reduction Reaction
Source: Nanomaterials (Basel). 2022 Dec 13;12(24):4432. doi: 10.3390/nano12244432 (PMC9782998; doi:10.3390/nano12244432)
Supplement: Supplementary file 1 [file nanomaterials-12-04432-s001.zip › nanomaterials-2102465-SI.pdf]

Supplementary Materials

Carbon Surface-Influenced Heterogeneity of Ni and Co Catalytic Sites as a Factor Affecting the Efficiency of Oxygen Reduction Reaction

Marc Florent and Teresa J. Bandosz *

Department of Chemistry and Biochemistry, The City College of The City University of New York, 160 Convent Avenue, New York, NY 10031, USA; mflorent@ccny.cuny.edu

* Correspondence: tbandosz@ccny.cuny.edu

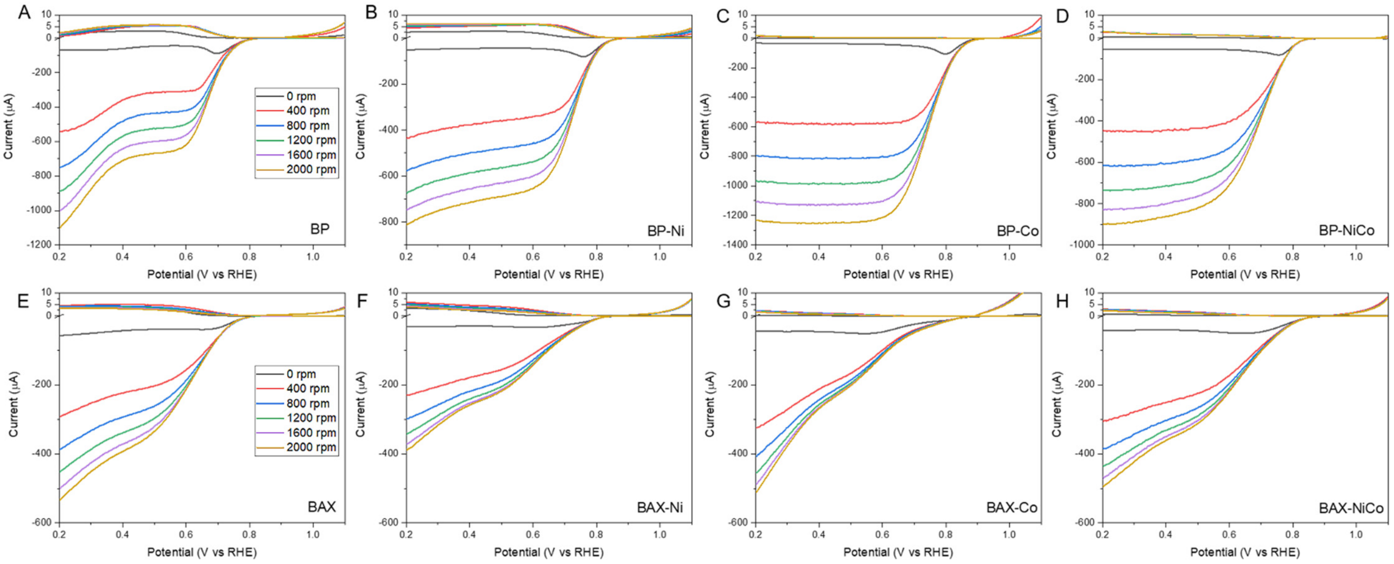

Figure S1. Ring and disk currents at different rotation (0-2000rpm) of the different samples: BP (**A**), BP-Ni (**B**), BP-Co (**C**), BP-NiCo (**D**), BAX (**E**), BAX-Ni (**F**), BAX-Co (**G**) and BAX-NiCo (**H**).

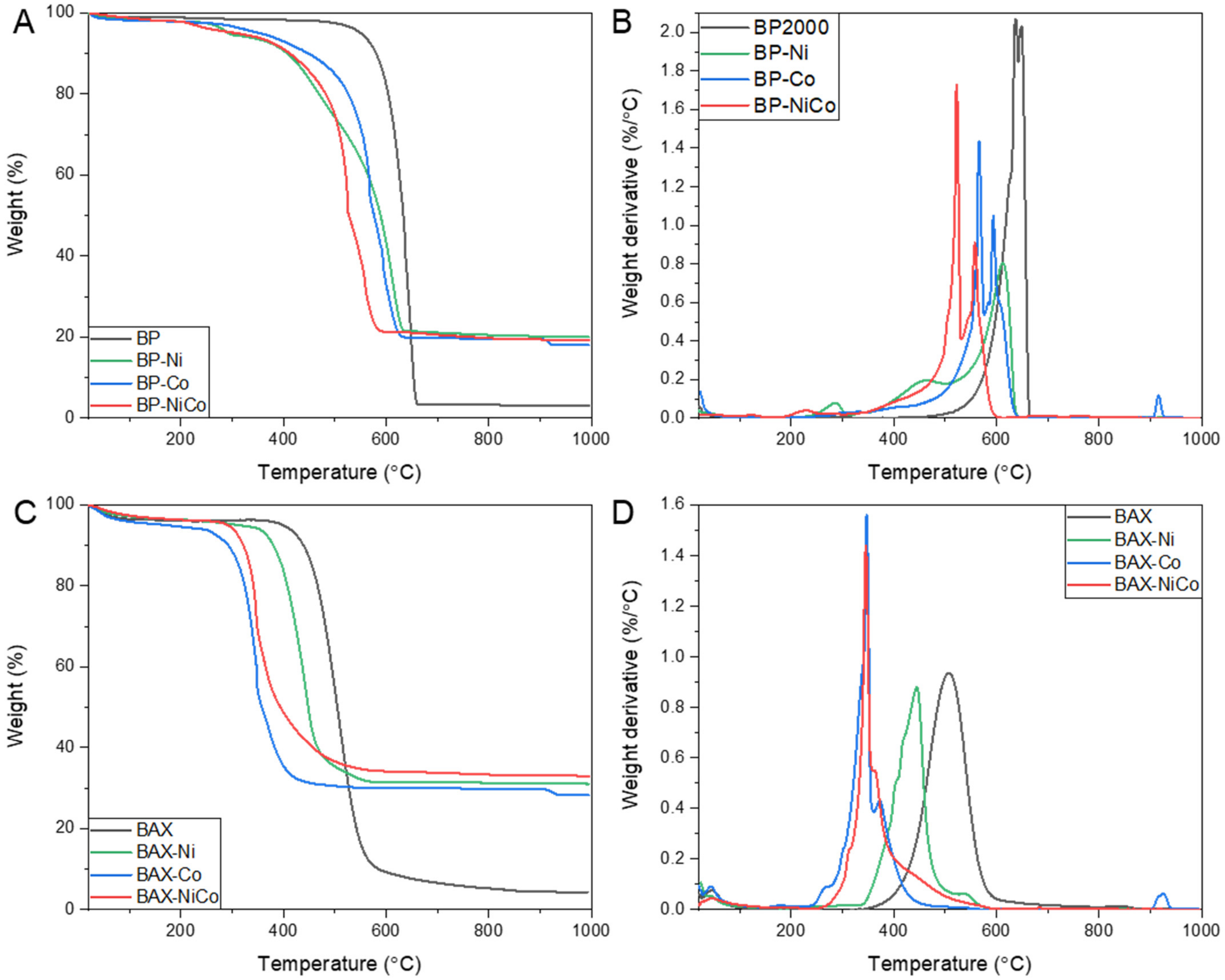

Figure S2. TG (**A, C**) and DTG (**B, D**) curves measured in air of the BP series (**A, B**) and of the BAX series (**C, D**).

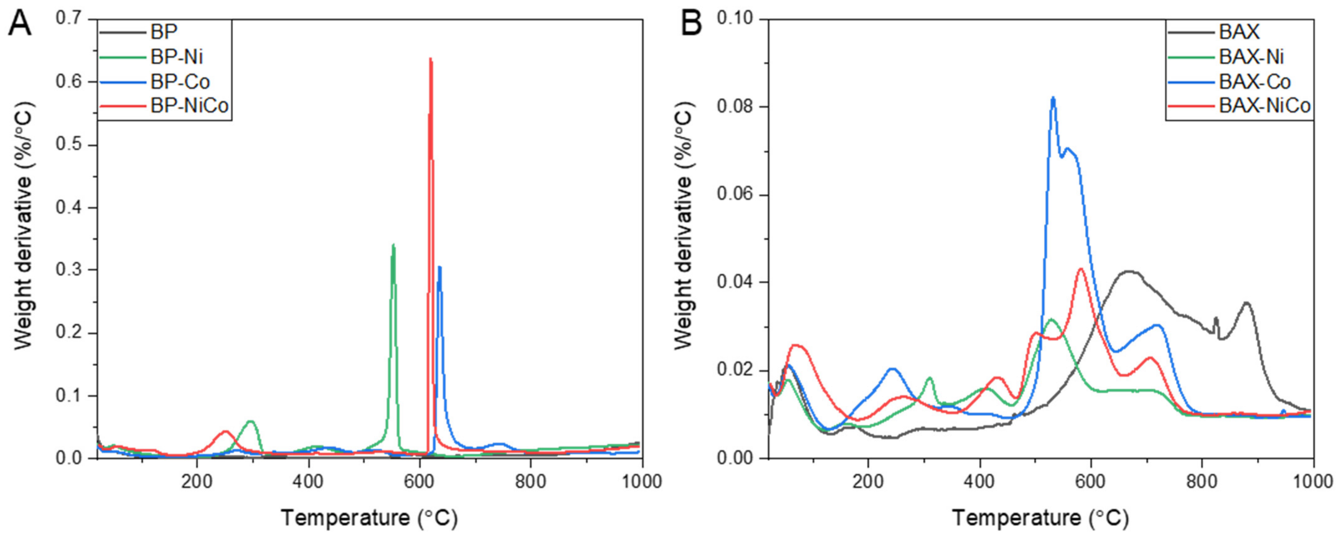

Figure S3. DTG curves measured in argon of the BP series (A) and of the BAX series (B).

Table S1. The atomic content of elements on the surface (in bold) and the deconvolution results of collected core energy level spectra

Binding energy (eV)	Chemical bonds	BP	BP-Ni	BP-Co	BP-NiCo	BAX	BAX-Ni	BAX-Co	BAX-NiCo
**C 1s**		**86.3**	**83.3**	**90.7**	**91.1**	**86.8**	**59.9**	**61.1**	**55.8**
284.7-284.9	C–C (sp^2)	77.0	74.9	75.7	64.2	73.8	69.3	76.5	66.9
286.0-286.5	C–N, C–O	16.0	17.7	17.8	19.5	13.4	14.3	11.6	15.7
287.1-287.6	C=O				7.6	5.3	6.9		7.5
288.5-289.2	C(O)=O	7.0	7.4	6.5	6.3	5.0	5.9	7.3	6.4
290.3-290.8	π–π*	–			2.4	2.6	3.6	4.5	3.5
**O 1s**		**12.3**	**11.3**	**7.5**	**6.2**	**13.2**	**28.6**	**27.8**	**32.9**
530.7-531.6	O-M		8.7	6.4	9.1		41.5	83.1	90.9
531.9-532.6	O=C	57.3	51.6	50.5	70.3	51.8	45.8	16.9	
533.5-533.8	O–C	42.7	39.7	43.1	20.6	48.2	12.7		9.1
**N 1s**		**1.4**	**0.9**	**1.0**	**–**	**–**	**–**	**–**	**–**
398.4-398.9	N=C	15.6							
399.1-399.3	N–H, N–C	73.3	59.0	32.5					
400.1	N^+	11.1	41.0	67.5					
**Ni 2p3/2**		**–**	**4.5**	**–**	**1.1**	**–**	**11.5**	**–**	**5.4**
	Ni		3.5		2.6		3.3		0.7
	NiO		41.8		27.1		39.5		4.1
	$Ni(OH)_2$		54.8		70.3		57.2		95.2
**Co 2p3/2**		**–**	**–**	**0.7**	**1.0**	**–**	**–**	**11.1**	**5.9**
	Co			-	4.2				1.4
	$Co(OH)_2$			18.1	66.6			64.1	54.6
	Co_3O_4			81.9	29.3			35.9	44.0
